# Dryland Wheat Domestication Changed the Development of Aboveground Architecture for a Well-Structured Canopy

**DOI:** 10.1371/journal.pone.0095825

**Published:** 2014-09-02

**Authors:** Pu-Fang Li, Zheng-Guo Cheng, Bao-Luo Ma, Jairo A. Palta, Hai-Yan Kong, Fei Mo, Jian-Yong Wang, Ying Zhu, Guang-Chao Lv, Asfa Batool, Xue Bai, Feng-Min Li, You-Cai Xiong

**Affiliations:** 1 State Key Laboratory of Grassland Agro-ecosystems, Institute of Arid Agroecology, School of Life Sciences, Lanzhou University, Lanzhou, China; 2 Eastern Cereal and Oilseed Research Centre (ECORC), Agriculture and Agri-Food Canada, Ottawa, Ontario, Canada; 3 CSIRO Plant Industry, Canberra, Australia; 4 School of Plant Biology, Faculty of Natural and Agricultural Sciences, The University of Western Australia, Crawley, Perth, Australia; China Agricultural University, China

## Abstract

We examined three different-ploidy wheat species to elucidate the development of aboveground architecture and its domesticated mechanism under environment-controlled field conditions. Architecture parameters including leaf, stem, spike and canopy morphology were measured together with biomass allocation, leaf net photosynthetic rate and instantaneous water use efficiency (WUE_i_). Canopy biomass density was decreased from diploid to tetraploid wheat, but increased to maximum in hexaploid wheat. Population yield in hexaploid wheat was higher than in diploid wheat, but the population fitness and individual competition ability was higher in diploid wheats. Plant architecture was modified from a compact type in diploid wheats to an incompact type in tetraploid wheats, and then to a more compact type of hexaploid wheats. Biomass accumulation, population yield, harvest index and the seed to leaf ratio increased from diploid to tetraploid and hexaploid, associated with heavier specific internode weight and greater canopy biomass density in hexaploid and tetraploid than in diploid wheat. Leaf photosynthetic rate and WUE_i_ were decreased from diploid to tetraploid and increased from tetraploid to hexaploid due to more compact leaf type in hexaploid and diploid than in tetraploid. Grain yield formation and WUE_i_ were closely associated with spatial stance of leaves and stems. We conclude that the ideotype of dryland wheats could be based on spatial reconstruction of leaf type and further exertion of leaf photosynthetic rate.

## Introduction

In arid and semi-arid regions, plants have evolved with large root systems for competing and adapting to water-limited environments as a result of natural selection [1]–[5]. Strong individual competitiveness for a crop species is closely related to more resources allocation into its vegetative organs, which improves the acquisition of resources such as water and nutrients, but reduces grain yield [6]–[8]. In the evolution of crop plants, natural selection promotes individual dominance, while breeding and selection require community superiority [6], [9]–[10]. Modern wheat crop cultivars have evolved with less individual competitiveness but high reproductive allocation and population yield through strenuous plant breeding effort.

High-yielding modern hexaploid wheats have a small root system with weak competition ability of individual plant and more resource allocation to reproductive growth, leading to an increased harvest index [6], [11]–[13]. Large root systems are generally associated with strong individual competition for water and nutrients, which in turn reduces crop population yield. On the other hand, leaves as major photosynthetic organs play a critical role in affecting assimilation production and source-sink relation. Since improving crop population yield is the goal in breeding and selection of cultivars, increasing leaf area is logically more important for production of photosynthetic assimilates and hence grain yield [14]–[19]. However, large leaves could lead to low light transmittance through the canopy affecting canopy photosynthesis and population yield [20]–[22].

Biomass accumulation is closely linked with the size of crop organs and their morphological construction [23]–[26]. It plays an important role in plant adaptation to adverse environments [27]–[29]. Previous studies on biomass accumulation in crop plants were mainly focused on the comparison of aboveground and underground biomass between wild and modern crops [28], [30]–[32]. Particularly, it was reported that the pattern of biomass accumulation in hexaploid wheat has been modified to have more photosynthetic assimilate investment and partitioning in the aboveground biomass, leading to smaller root-shoot ratios [28], [33]. Relevant studies on the relationship between the pattern of biomass accumulation and the morphological change in photosynthetic organs during the domestication of wheats were critical. Since Donald proposed the definition of crop ideotype in 1968, great efforts have been made to explore ideotype of dryland wheat and its evolvement strategy; however little progress was achieved [6], [8]–[10], [14], [56].

Our previous studies indicated that dryland wheats evolved towards the trend of increasing drought tolerance, greater water use efficiency and population grain yield. In those studies, we used 6 wheat varieties representing the domestication route from diploid to tetraploid and further hexaploid wheat cultivars [28]. Among these cultivars, MO1 and MO4 are diploid (*Triticum monococcum* L.) species (einkorn wheat) with AA genome. The cultivation of einkorn wheat can be traced back to the Bronze Age. DM22 and DM31 (emmer) belong to tetraploid wheats (*Triticum dicoccum* Schuebl L.) with AABB genome, which originated from diploid species. Two hexaploid wheat (*Triticum aestivum* L.), cultivars L8275 and Monkhead (AABBDD) originated from tetraploid genome cross with wild diploid species (*Aegilops tauschii*) [34]–[39]. The A genome of *Triticum monococcum* L. and the A and B genomes of *Triticum dicoccum* Schuebl L. are evolutionally homologous with those of *Triticum aestivum* L. [40]. Domestication of einkorn and emmer wheat was involved the transition from hunting and gathering to cultivation of wild plants about 10,000 years ago. During the long-term domestication of dryland wheats, hard and bread wheats were selected and remained, in which tetraploid *T. durum* became the widely cultivated wheat crop today, and bread wheat gradually became the world's leading crop [38]–[39].

The aims of this study were to determine the changes in the partitioning of biomass between plant parts, the architecture of leaves and stems and of leaf photosynthesis and transpiration rates as a result of natural and artificial selection in different ploid wheats. Quantitative analyses including analysis of variance (ANOVA), biplot analysis by using ‘tester standard deviation’ scaling and ‘GGE’ model were made to assess the plant type evolvement and its ecological implication in breeding strategic innovation. We hypothesized that biomass partitioning pattern was a critical factor to drive adaptive changes in leaf/canopy architecture, which accordingly resulted in the changes in leaf photosynthetic rate and yield formation during the domestication process of dryland wheats. The results would provide new understandings on dryland wheats adaptation and their breeding strategies.

## Materials and Methods

### Plant Material and Growth Conditions

Six genotypes (two diploids, T. *monococcum* L., MO1 and MO4; two tetraploids, T. *dicoccum* Schuebl, DM22 and DM31; two commercial hexaploid wheats) were chosen in this study (Table 1). These genotypes were used to represent *Triticum* species with three ploidy levels and genome sizes. These wheat genotypes to some extent can reflect a relatively certain route of dryland wheat domestication (Table 1). Tetraploid hard wheat and hexaploid bread wheat represent the final steps in *Triticum* domestication, whereas DM22 and DM31 are non-free-threshing wheat genotypes which belong to older genotypes than hexaploid L8275 and Monkhead [38]–[39]. The seeds of six genotypes were procured from Chinese Crop Germplasm Resources Database (administered by the Institute of Crop Germplasm Resources, Chinese Academy of Agricultural Sciences, Beijing, China).

**Table 1:** List of diploid, tetraploid and hexaploid wheats and their characteristics grown at Yuzhong Experimental Station of Lanzhou University, China in 2009 and 2010.

Ploidy	Genotypes	Chromosome type	Genotype properties
Diploid	MO1	AA	*T.monococcum* L.
	MO4	AA	*T.monococcum* L.
Tetraploid	DM22	AABB	*T.dicoccum Schuebl.*
	DM31	AABB	*T.dicoccum Schuebl.*
Hexaploid	*Monkhead*	AABBDD	Old cultivar
	*Longchun8275*	AABBDD	Modern cultivar

The field experiment was conducted for two years (from 21 March to 26 July 2009, and 23 March–27 July 2010) at Yuzhong Experimental Station of Lanzhou University in Yuzhong County of Gansu Province (35°56′34.47″N, 104°08′49.35″E), China. This site is representative of the semi-arid climate in northwestern China, with long-term average of 330 mm annual rainfall, 1,700 mm evaporation, 14.5°C mean temperature, and 58% relative humidity during the growing season (March–July). The experimental field had a Heima sandy loam soil (Calcic Kastanozems, FAO Taxonomy), with 26.6% field water capacity and 5.8% permanent wilting point [41].

In each year, the experimental field was applied with 120 kg ha^−1^ of N as urea, 60 kg ha^−1^ of P as amended superphosphate and 48 kg ha_−1_ of K potash before planting. The chosen genotypes of diploid, tetraploid and hexaploid wheats were sown in a randomized complete block design with three replications. Each plot was 2.0 m long and comprised 6 rows with row spacing of 0.2 m. Before seeding, the seeds were vernalized at 4° for one week. The plots were sown by hand at a seed rate of 250 seeds m^−2^ at the end of March each year. Weeds were controlled by hoeing according to local practices.

### Measurements

At flowering stage, plant growth parameters (leaf angle, leaf area, tiller number, root biomass, stem biomass, spike biomass) were monitored. At physiological maturity, seed yield per plant and population yield were determined.

### Leaf Traits

**Figure 1:**
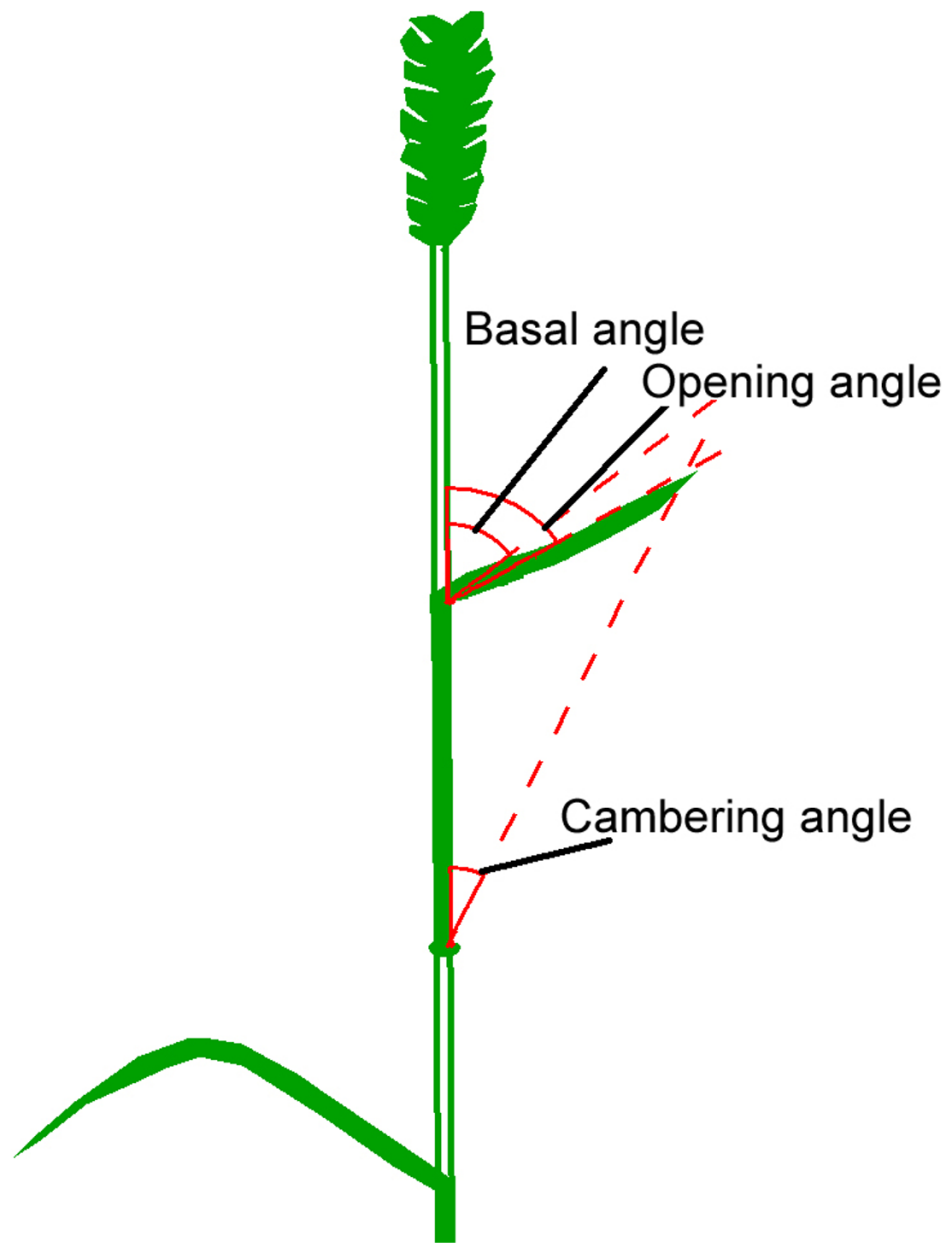
Diagrammatic representation of the basal angle (the angle between the base of a leaf and the upper stem of the leaf), opening angle (the angle between the tip of the leaf blade and the stem) and cambering angle (the angle between the tip of the leaf blade and the upper stem of the leaf position).

At flowering stage, thirty representative plants from the central four rows in each plot were selected and labeled for characterizing leaf architecture. Spatial angles of the top three leaves (i.e. the flag leaf, penultimate leaf and the 3rd leaf from the top), including the basal angle (the upper (adaxial) angle between a leaf and a stem), splaying angle (the angle between the line connecting leaf blade tip and the stem) and cambering angle (the angle between the leaf blade tip and the upper stem of leaf position) (Fig. 1), were determined from 8:00 to 11:00 am with a protractor and repeated five times. Leaf length, leaf width, leaf photosynthetic rate and transpiration rate of the same leaves were determined concurrently. Leaf area was calculated according to the formula: leaf area = length×width×0.83 (Xiong et al., 2006). Leaf photosynthetic rate and transpiration rate were measured using a portable LI-COR Li-6400/XT gas exchange system (LI-COR, Lincoln, NE, USA), between 9:00 and 11:00 am when the day was clear and sunny. The photosynthetic photon flux density (PAR) during the measurements was maintained at 1500 µmol m-2 s-1 using a LI-COR LI-6400-02B red-blue light source. Water use efficiency (WUEi) was calculated as WUEi = Photosynthesis rate/Transpiration rate.

All the leaf traits were measured in 2009, and the leaf architecture traits (leaf basal angle, leaf splaying angle, leaf cambering angle, leaf length and leaf width) were also repeated in 2010.

### Stem Traits, Plant Dry Weight, Yield and Yield Components

At physiological maturity, the predetermined 30 plants of each plot were used to characterize the color of the uppermost internode [26], plant height (from the ground surface to the top of the spike, excluding the awns), leaf location (from ground to leaf orientation) and number of tillers per plant. The plants were carefully harvested by hand and separated each leaf, each internode and spike. Node width and internode length were measured by vernier caliper and ruler, kernels per spike were also determined. The individual plant components were bagged and oven–dried at 80°C for 48 h and recorded for dry weight. Spikes were then threshed and grains were re-dried at 40°C for 24 h to record the dry weight. Population yield was measured from the four central rows of each plot, including the thirty plants (experiment samples) per plot. All dry matter data were reported at 0 water basis.

Roots (0–160 cm) were collected by a root drill, soaked in a pail and thoroughly cleaned. These root samples were also been oven-dried at 80°C for dry weight.

All these above traits were determined in 2009's experiment, while tiller number, root biomass, stem biomass, spike biomass, yield per plant and population yield were measured in both years.

Some relative indexes were calculated according to the following equations [21]:

1. Leaf length-width Ratio = leaf length/leaf width
2. Flag leaf length-width Ratio (FLWR) = flag leaf length/flag leaf width
3. Internode specific gravity (SGN) = internode weight/(internode length×internode diameter^2^)
4. Internode weight-diameter ratio (RWD) = internode length/internode dry weight
5. Leaf specific weight (SLW) = leaf dry weight/leaf area
6. Leaf sheath length-internode length ration = leaf sheath length/internode length
7. Internode length – plant height ratio = internode length/plant height
8. Grain-leaf ratio = grains per spike/leaf area
9. Spike-leaf ratio = dry matter of ear/leaf area
10. Plant height-ear Ratio (HER) = Plant height/ear length
11. Harvest index (HI) = Grain yield/total biomass (shoot mass + root mass)

### Statistical Analyses

Analysis of variance (ANOVA) was performed for all variables; these traits were compared by least significant difference (LSD) at the 0.05 level of probability. ANOVA residuals were used for the calculation of the 5% LSD; this was done under the assumption of homogeneity of variances (Levene test). Principal component analysis (PCA) of the main characteristics (plant height (PH), height-ear ratio (HER), leaf area (LA), flag leaf length-width ratio (FLWR), second leaf length-width ratio (SLWR), total leaf area (LE), photosynthetic rate of flag leaf (PR), tiller number (TN), length of fifth internode (FNL), first node width (ND), position of flag leaf (LL), ear length (EL), were performed and illustrated using GGE biplot. Then different plant traits (PC1) and different diploid wheat (PC2) were acted as the first principal component and the second principal component. The GGE Biplot allowed us to examine the relationship between different diploid wheat and different plant traits by direction of different vector and the angle between vectors.

## Results

### Leaf Architecture Domestication among Different Ploidy Wheats

Analysis of variance (ANOVA) was performed to reveal leaf type traits of flag leaf, penultimate leaf and the 3^rd^ leaf (three major functional leaves) from diploid to tetraploid, tetraploid to hexaploid and diploid to hexaploid wheats (Table 2). It was found that all the parameters including leaf spatial angles and relevant morphological traits generally significantly increased from diploid to tetraploid wheats, but there were no significant changes from tetraploid to hexaploid wheats (Table 2). Specifically, the changes in leaf type varied from morphological parameters and wheat genotypes. Specific leaf weight (SLW) remained unchanged among three ploid wheats (Table 2). The basal, opening and cambering angles of leaf (refer to three functional leaves) were wholly significantly increased from diploid to tetraploid wheats (*p*<0.05), suggesting that spatial stance of leaves tended to become more incompact during this transition. Leaf length, leaf width, leaf area, leaf weight and leaf sheath length also increased, however leaf length-width ratio was reduced significantly from diploid to tetraploid wheats (Table 2).

**Table 2:** Changes in the characteristics of leaf type traits from diploid to tetraploid, tetraploid to hexaploid and diploid to hexaploid wheats.

Leaf characteristics	Leaf type	Diploid old wheat	Tetraploid old wheat	Hexaploid moden wheat	Diploid to Tetraploid	Tetraploid to Hexaploid	Diploid to Hexaploid
Basal angle (°)	Flag	22.1±1.6	30.0±2.0	21.0±1.9	+	−	0
	Penultimate	25.1±1.3	33.0±2.1	24.2±2.0	+	−	0
	Top 3^rd^	22.7±1.6	34.6±3.1	23.6±2.6	+	−	0
Opening angle (°)	Flag	30±1.8	65.4±3.8	46.0±2.3	+	−	+
	Penultimate	39.4±2.1	66.0±6.1	60.6±5.9	+	0	+
	Top 3^rd^	40.3±3.2	60.1±5.2	53.0±3.6	+	0	+
Cambering angle (°)	Flag	19.0±1.3	27.4±1.9	18.2±1.5	+	−	0
	Penultimate	25.3±2.2	36.2±2.6	22.1±3.2	+	−	0
	Top 3^rd^	24.3±2.1	36.4±3.1	22.9±2.6	+	−	0
Length (cm)	Flag	14.1±1.2	18.5±1.2	22.0±1.2	+	−	+
	Penultimate	18.2±1.6	22.5±1.0	22.5±1.2	+	0	+
	Top 3^rd^	16.2±1.1	21.9±2	22.1±1.4	+	0	+
Width (cm)	Flag	0.8±0.02	1.4±0.05	1.39±0.05	+	0	+
	Penultimate	0.8±0.01	1.2±0.03	1.2±0.04	+	0	+
	Top 3^rd^	0.6±0.02	1.0±0.1	1.0±0.1	+	0	+
Area (cm^2^)	Flag	9.5±0.4	21.8±1.5	23.2±1.8	+	0	+
	Penultimate	12.6±0.8	22.9±1.2	23.4±1.6	+	0	+
	Top 3^rd^	8.6±0.8	18.6±2.2	18.2±1.5	+	0	+
Length-width ratio	Flag	17.6±2.7	15.3±0.8	13.7±0.5	−	−	−
	Penultimate	22.2±1.8	18.7±0.9	18.5±0.7	−	0	−
	Top 3^rd^	26.5±1.2	21.8±1.8	23.1±2.1	−	0	−
Specific leaf weight (g/cm^2^)	Flag	3.1±0.3	3.3±0.2	3.5±0.5	0	0	0
	Penultimate	3.0±0.3	2.8±0.2	3.0±0.2	0	0	0
	Top 3^rd^	3.0±0.1	2.8±0. 3	2.9±0.4	0	0	0
Sheath length (cm)	Flag leaf	14.3±1.8	22.4±0.9	19.2±1.1	+	−	+
	Penultimate	10.5±1.5	16.8±1.3	16.0±1.7	+	0	+
	Top 3^rd^	9.1±0.7	13.5±1.3	13.4±1.6	+	0	+

+ represents a significantly increase at P<0.05. − represents a significant decrease at P<0.01. 0 represents no change. The data of Leaf Basal angle and Opening angle Cambering angle and leaf area were similar in 2009–2010 and average data are presented in the table.

Note: The table involved large data, here we showed the final results for clarity and conciseness.

On the other hand, the basal and cambering angles of leaf were significantly decreased from tetraploid to hexaploid wheats. In addition, there were no significant changes in leaf width and leaf area for all three functional leaves. Moreover, no significant changes were observed in leaf opening angle, leaf length, leaf length-width ratio and leaf sheath length in the penultimate and the 3^rd^ leaves, except that significant decrease was observed in flag leaf. This showed that flag leaf may play more sensitive role in the evolvement of leaf type than other leaves, and spatial stance of leaves tended to become more compact in hexaploid wheats in comparison with that of diploid wheats (Table 2).

### Leaf Physiological Domestication among Ploidy Wheats

Leaf net photosynthesis rates of flag leaf, penultimate leaf and the 3^rd^ leaf were decreased significantly from diploid to tetraploid, but were increased significantly from tetraploid to hexaploid (Table 3). In addition, transpiration rates of these major functional leaves increased significantly from diploid to tetraploid, but were decreased from tetraploid to hexaploid. No significant differences in transpiration rate were observed between diploid and hexaploid wheats (Table 3). Furthermore, the instantaneous WUE_i_ followed a similar trend as did in leaf net photosynthesis rate, i.e. the WUE_i_ increased from diploid to tetraploid but was decreased from tetraploid to hexaploid. It was noted that WUEi had a significant reduction from diploid to hexaploid wheats (Table 3). The results indicated that leaf photosynthetic rate might have an internal coupling relationship with leaf transpiration rate during the domestication of dryland wheats from diploid to tetraploid and hexaploid.

**Table 3:** Changes in leaf net photosynthetic rate, transpiration rate and the instantaneous water use efficiency (WUE_i_ = leaf photosynthetic rate/transpiration rate) of the flag leaf, penultimate and 3^rd^ leaves in the canopy in the domestication from diploid to tetraploid, tetraploid to hexaploid and diploid to hexaploid of wheats.

Physiological characteristics	Leaf type	Diploid old wheat	Tetraploid old wheat	Hexaploid moden wheat	Diploid to tetraploid	Tetraploid to hexaploid	Diploid to hexaploid
Leaf photosynthetic rate(µmol m^−2^ s^−1^)	Flag	18.6±0.6	12.3±0.6	13.8±0.4	−	+	−
	Penultimate	12.1±0.3	8.7±0.4	9.5±0.4	−	+	−
	Top 3^rd^	4.9±o.2	3.2±0.2	4.1±0.3	−	+	−
Leaf transpiration rate (mmol m^−2^ s^−1^)	Flag	6.1±0.7	8.8±0.6	5.5±0. 4	+	−	0
	Penultimate	5.6±0.4	7.4±0.5	5.1±0.3	+	−	0
	Top 3^rd^	2.2±0.1	2.9±0.2	2.3±0.1	+	−	0
WUE_i_	Flag	3±0.2	1.4±0.1	2.4±1.6	−	+	−
	Penultimate	2.2±0.6	1.2±0.2	1.8±0.3	−	+	−
	Top 3^rd^	2.2±0.2	1.1±0.1	1.7±0.4	−	+	−

+ represents a significant increase at P<0.01. − represents a significant decrease at P<0.01. 0 represents no change.

Note: The table involved large data, here we showed the final results for clarity and conciseness.

### Stem, Spike and Tiller Architecture Domestication among Different Ploidy Wheats

To compare morphological differences in supporting organ (stem) and reproductive organ (spike) among six wheat genotypes, a group of critical parameters including stem internode, plant height, tiller number and spike were measured and analyzed (Table 4). Along with the increase in ploidy of the chromosome numbers from diploid to tetraploid and hexaploid, number of tillers per plant was decreased while spike length increased (Table 4). Awn length also increased from diploid to tetraploid, but it was decreased from tetraploid to hexaploid (Table 4). Reduction in plant height from diploid to tetraploid was caused mainly by the extension of the 5^th^ internode. In contrast, from tetraploid to hexaploid, plant height remained unchanged with slightly longer first 4 internodes and shorter 5^th^ internode (Table 4). Importantly, the diameter of stem increased significantly from diploid to tetraploid, but did not change from tetraploid to hexaploid (Table 4). Internode weight-diameter ratio (RWD) also increased significantly from diploid to tetraploid and then to hexaploid, except for the 4^th^ and the 5^th^ internodes, which remained unchanged from tetraploid to hexaploid (Table 4). In addition, internode specific gravity (SGN) did not change among all six wheat genotypes (Table 4). It can be found that supporting organ (stem) tended to become thicker and higher while reproductive organs became well-structured. In the mean time, tillering ability was significantly weakened with the development of chromosome ploidy.

**Table 4:** Changes in plant height, tiller number, spike length, awn length and internode characteristics of nodes 1 to 5 in the domestication from diploid to tetraploid, tetraploid to hexaploid and diploid to hexaploid of wheats.

Stem characteristics	Internode type	Diploid old wheat	Tetraploid old wheat	Hexaploid modern wheat	Diploid to tetraploid	Tetraploid to hexaploid	Diploid to hexaploid
Plant height (cm)		105.1±4.5	120.4±7.9	127.3±6.5	+	0	+
Tiller number		8.6±1.0	4.8±0.4	1.9±0.2	−	−	−
Internode length (cm)	1^st^	5.1±0.4	5.2±0.4	8.5±0.8	0	+	+
	2^nd^	11.4±1.8	11.5±1.6	15.0±2.0	0	+	+
	3^rd^	15.1±1.3	16.1±1.7	21.5±4.1	0	+	+
	4^th^	22.5±2.5	22.5±2.6	28.2±3.1	0	+	+
	5^th^	39.8±6.1	49.5±4.2	42.1±3.7	+	−	0
Internode diameter (mm)	1^st^	1.6±0.2	2.6±0.3	2.7±0.2	+	0	+
	2^nd^	2.0±0.2	3.0±0.2	3.2±0.3	+	0	+
	3^rd^	2.2±0.1	3.3±0.3	3.4±0.5	+	0	+
	4^th^	2.1±0.3	3.5±0.3	3.6±0.3	+	0	+
	5^th^	1.7±0.3	2.7±0.2	2.7±0.1	+	0	+
Internode weight-diameter ratio (g/mm)	1^st^	0.03±0	0.05±0.002	0.06±0.001	+	+	+
	2^nd^	0.05±0.002	0.08±0.003	0.09±0.005	+	+	+
	3^rd^	0.04±0.003	0.08±0.003	0.10±0.003	+	+	+
	4^th^	0.06±0.002	0.1±0.01	0.10±0.006	+	0	+
	5^th^	0.07±0.003	0.13±0.003	0.12±0.01	+	0	+
Internode specific gravity (g*mm^−10^)	1^st^	4.8±0.5	4.1±0.6	4.2±0.3	0	0	0
	2^nd^	2.2±0.1	2.3±0.2	2.0±0.2	0	0	0
	3^rd^	1.3±0.2	1.5±0.4	1.4±0.2	0	0	0
	4^th^	1.2±0.2	1.4±0.2	1.2±0.3	0	0	0
	5^th^	1.2±0.1	1.1±0.1	1.0±0.1	0	0	0
Spike (cm)	Length	4.5±0.2	7.2±0.3	9.0±0.7	+	+	+
	Awn length	7.0±0.7	9.5±2.1	6.7±0.9	+	−	0

+ represents a significant increase at P<0.01. − represents a significant decrease at P<0.01. 0 represents no change. Results of tiller number were similar between 2009 and 2010, and average data are presented in the table.

Note: The table involved large data, here we showed the final results for clarity and conciseness.

### Canopy Architecture Domestication among Ploidy Wheats

A few critical parameters of canopy architecture were compared. The spatial position of the flag leaf, penultimate and the 3^rd^ leaves shifted upward from diploid to tetraploid and hexaploid (Table 5). The ratio of spike length to plant height had the same trend (Table 5). The ratio of leaf sheath length to internode length increased fFrom diploid to tetraploid but was decreased from tetraploid to hexaploid in the upper four leaves, except for the flag leaf sheath. We also compared the variation in the ratio of internode length to plant height in the upper five internodes. Our data showed that hexaploid wheats had more advantages in this ratio. Greater ratio resulted from the elongation of upper three internodes but not from the lower 4^th^ and 5^th^ internodes (Table 5).

**Table 5:** Morphological changes in the canopy through the domestication from diploid to tetraploid, tetraploid to hexaploid and diploid to hexaploid of wheats.

Canopy morphology		Diploid old wheat	Tetraploid old wheat	Hexaploid modern wheat	Diploid to tetraploid	Tetraploid to hexaploid	Diploid to hexaploid
Spike length - plant height ratio		0.04±0.001	0.06±0.003	0.07±0.002	+	+	+
Location of leaf (cm)	Flag leaf	67.0±2.2	74.2±1.6	90.0±4.2	+	+	+
	Penultimate leaf	41.2±3.2	47.1±1.3	55.5±3.5	+	+	+
	Top 3^rd^ leaf	25.0±1.8	28.3±1.1	33.3±1.6	+	+	+
	Top 4^th^ leaf	12.6±3.8	14.7±3.1	16.0±2.6	0	0	0
Leaf sheath length-node length ratio	Flag leaf	0.37±0.01	0.47±0.02	0.49±0.02	+	0	+
	Penultimate leaf	0.47±0.01	0.76±0.04	0.60±0.03	+	−	+
	Top 3^rd^ leaf	0.60±0.01	0.85±0.03	0.65±0.03	+	−	+
	Top 4^th^ leaf	0.69±0.02	0.97±0.04	0.8±0.04	+	−	+
Internode length - plant height ratio	1^st^ internode	0.04±0.004	0.04±0.003	0.07±0.006	0	+	+
	2^nd^ internode	0.11±0.001	0.09±0.003	0.11±0.004	−	+	0
	3^rd^ internode	0.14±0.003	0.14±0.01	0.17±0.007	0	+	+
	4^th^ internode	0.21±0.01	0.19±0.005	0.22±0.01	−	+	0
	5^th^ internode	0.38±0.01	0.41±0.02	0.33±0.03	+	−	−

+ represents a significant increase at P<0.01. − represents a significant decrease at P<0.01. 0 represents no change.

Note: The table involved large data, here we showed the final results for clarity and conciseness.

### Biomass Distribution and Source-Sink Relation among Different Ploidy Wheats

Biomass distribution pattern plays a critical role in affecting plant type and source-sink relation. The data showed that leaf weight, stem weight, internode weight, leaf sheath weight and total biomass all increased from diploid to tetraploid, but remained unchanged from tetraploid to hexaploid (Table 6). Spike biomass and aboveground biomass increased significantly from diploid to tetraploid and then to hexaploid (Table 6). Root biomass was decreased significantly along with the increase in chromosome ploidy from diploid to tetraploid and hexaploid (Table 6). Furthermore, a few critical ratios of biomass distribution were calculated and compared. It suggested that grain-to-leaf ratio remained unchanged from diploid to tetraploid, but increased significantly from tetraploid to hexaploid (Table 6). Spike-leaf ratio was decreased significantly from diploid to tetraploid, but increased from tetraploid to hexaploid. More importantly, harvest index increased steadily along with the increase in chromosome ploidy, from diploid to tetraploid and hexaploid (Table 6).

**Table 6:** Changes in the pattern of biomass distribution through the domestication from diploid to tetraploid, tetraploid to hexaploid and diploid to hexaploid for wheats.

Biomass distribution pattern		Diploid old wheat	Tetraploid old wheat	Hexaploid modern wheat	Diploid to tetraploid	Tetraploid to hexaploid	Diploid to hexaploid
Leaf dry weight (g)	Flag leaf	0.03±0.001	0.07±0002	0.07±0.003	+	0	+
	Penultimate Leaf	0.04±0.002	0.06±0.003	0.06±0.003	+	0	+
	Top 3^rd^ leaf	0.02±0.002	0.04±0.002	0.04±0.002	+	0	+
	Top 4^th^ leaf	0.02±0.001	0.03±0.002	0.3±0.002	+	0	+
Leaf sheath dry weight (g)	Flag leaf	0.06±0.004	0.19±0.01	0.18±0.02	+	0	+
	Penultimate leaf	0.05±0.003	0.12±0.009	0.12±0.01	+	0	+
	Top 3^rd^ leaf	0.03±0.002	0.07±0.006	0.08±0.01	+	0	+
	Top 4^th^ leaf	0.02±0.009	0.04±0.002	0.04±0.002	+	0	+
Internode dry weight (g)	1^st^ internode	0.05±0.006	0.12±0.01	0.16±0.01	+	+	+
	2^nd^ internode	0.09±0.005	0.22±0.02	0.30±0.03	+	+	+
	3^rd^ internode	0.10±0.02	0.27±0.02	0.36±0.03	+	+	+
	4^th^ internode	0.13±0.01	0.34±0.02	0.37±0.03	+	0	+
	5^th^ internode	0.13±0.01	0.34±0.02	0.31±0.03	+	0	+
Total stem biomass (g)		0.52±0.04	1.3±0.2	1.5±0.3	+	0	+
Total leaf biomass (g)		0.11±0.02	0.21±0.03	0.23±0.04	+	0	+
Total leaf sheath biomass (g)		0.17±0.009	0.42±0.03	0.43±0.03	+	0	+
Total spike biomass (g)		0.71±0.02	1.10±0.04	1.72±0.03	+	+	+
Above-ground biomass (g)		1.51±0.01	3.02±0.03	3.88±0.04	+	+	+
Root biomass (g)		1.15±0.06	0.80±0.04	0.63±0.04	−	−	−
Total biomass (g)		2.65±0.08	3.83±0.7	4.52±0.62	+	0	+
Grain-leaf ratio (Grains/cm^2^)	Flag leaf	53.5±5.0	58.1±3.2	71.7±7.3	0	+	+
	Penultimate leaf	40.4±3.5	44.8±5.9	62.2±5.6	0	+	+
	Top 3^rd^ leaf	56.9±4.5	55.6±4.1	76.9±4.2	0	+	+
	Total leaf	15.8±4.2	15.2±2.2	22.5±2.5	0	+	+
Spike-leaf ratio (g/cm^2^)	Flag leaf	79.4±6.9	48.2±4.8	91.3±8.3	−	+	+
	Penultimate leaf	60.5±4.9	50.3±4.3	79.4±3.9	−	+	+
	Top 3^rd^ leaf	85.4±6.4	63.7±6.5	98.5±5.1	−	+	+
	Total leaf	23.7±1.8	17.3±1.6	28.8±2.4	−	+	+
Harvest index		0.23±0.01	0.30±0.02	0.40±0.01	+	+	+

+ represents a significant increase at P<0.01. − represents a significant decrease at P<0.01. 0 represents no remarkable change. Results of root biomass, stem biomass, and spike biomass were similar in 2009 and 2010, and average data are presented in the table.

Note: The table involved large data, here we showed the final results for clarity and conciseness.

### Yield Formation in Different Ploidy Wheats

Crop yield formation is widely recognized as a result of biomass distribution and system development. We compared the differences in grain weight per spike, grain number per spike, 1000-grain weight, spike number per plant, grain yield per plant and population yield. The results indicated that both grain weight per spike and grain number per spike increased significantly (*p*<0.05) from diploid to tetraploid to hexaploid (Table 7). Yet, the 1000-grain weight increased from diploid to tetraploid, but it did not change from tetraploid to hexaploid (Table 7). Particularly, both grain yield per plant and population grain yield also increased from diploid to tetraploid and hexaploid (Table 7).

**Table 7:** Changes in the grain yield and yield components through the dometication from diploid to tetraploid, tetraploid to hexaploid and diploid to hexaploid for wheats.

Grain yield components	Diploid old wheat	Tetraploid old wheat	Hexaploid modern wheat	Diploid to tetraploid	Tetraploid to hexaploid	Diploid to hexaploid
Grain weight/spike (g)	0.17±0.01	0.45±0.03	0.96±0.04	+	+	+
Grain number/spike	13.2±0.8	32.5±2.2	33.8±1.7	+	+	+
1000-grain weight (g)	36±4.1	42.4±3.6	41.7±3.1	+	0	+
Spike number/plant	5.4±0.2	3.2±0.2	1.9±0.2	−	−	−
Grain yield/plant (g)	0.47±0.04	0.96±0.05	1.83±0.08	+	+	+
Population yield (g/m^2^)	97.5±10.2	235.6±11.8	467.6±12.3	+	+	+

+ represents a significant increase at P<0.01. − represents a significant decrease at P<0.01. 0 represents no change. Data of yield per plant and population yield were similar in 2009 and 2010, and average data are presented in the table.

### The Changes in Yield-related Traits in Different Ploidy Wheats

**Figure 2:**
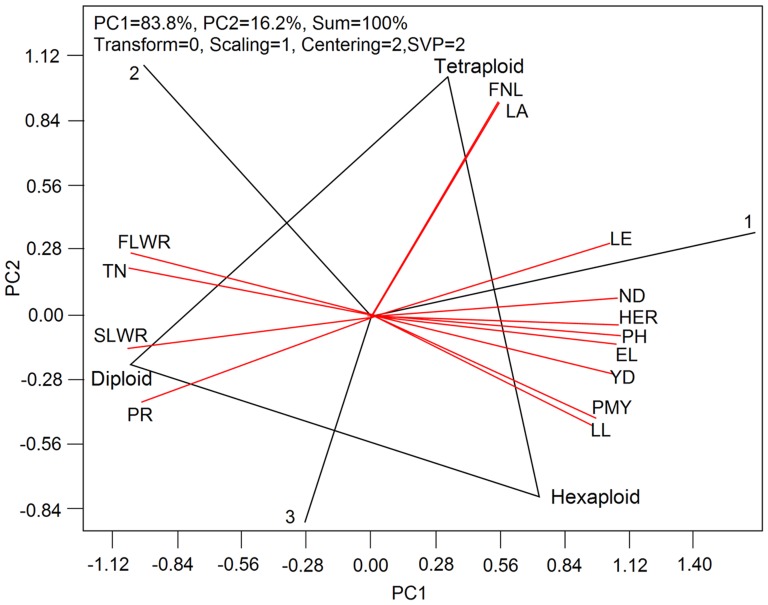
The biplot generated by using ‘tester standard deviation’ scaling and ‘GGE’ model. The biplot is based on different diploid wheat-centered (Centering = 2) and scaled data (Scaling = 1), using different diploid wheat -focused singular value partitioning (SVP = 2) method. YD, yield; PH, plant height; HER, height-spike ratio; LA, flag leaf angle; FLWR, the length-width ratio of flag leaf; SLWR, the length-width ratio of second leaf; LE, the total leaf area; PR, the photosynthetic rate of flag leaf; TN, tiller number; FNL, the length of fifth internode; ND, the first internode diameter; LL, the position of flag leaf; EL, spike length.

To comprehensively evaluate quantitative relationship between yield formation and canopy structure, we made a biplot analysis using ‘tester standard deviation’ scaling and ‘GGE’ model as illustrated in Figure 2. It showed that phenotypic traits of aboveground parts had different contribution to yield formation in the process of dryland wheat domestication. Diploid wheats had more advantages in length-width ratio of flag leaf (FLWR), tiller number (TN), length-width ratio of second leaf (SLWR) and photosynthetic rate of flag leaf (PR). With the doubling of chromosome number, tetraploid wheats had the highest flag leaf angle (LA) and the greatest length of fifth internode (FNL). However for other critical yield-related traits, hexaploid wheats had a superiority in individual yield (YD), population yield (PMY), total leaf area (LE), first internode diameter (ND), height-spike ratio (HER), plant height (PH), spike length (EL) and the position of flag leaf (LL) (Figure 2).

## Discussion

Our experimental data illustrated that natural selection and early domestication has modified wheat plant architecture from compact stance of diploid wheats to incompact stance of tetraploid wheats. Artificial domestication has resulted in more compact plant architecture in modern hexaploid wheats. The increase in architecture incompactness from diploid to tetraploid was mainly associated with substantial changes in leaf basal angle, leaf opening angle and leaf cambering angle. It was observed that leaf angles described above all increased from diploid to tetraploid wheats. Leaf basal angles and leaf cambering angle were decreased from tetraploid to hexaploid wheats, suggesting that canopy architecture was shifted from incompact stance to compact stance. Besides, leaf opening angle increased from diploid to hexaploid wheats. The wider leaf opening angle in the tetraploid and hexaploid wheat was likely a consequence of substantial increase in leaf area, presumably because more space was required to accommodate larger leaves in the canopy [42]. On the other hand, the increases in leaf length and leaf width led to overall expansion of leaf area in top leaves particularly in the penultimate and the third top leaves in the canopy.

Differences in light and space competition between aboveground organs were not measured in this study. It was assumed that the changes in leaf morphology had lowered the intensity of competition among aboveground organs, and accordingly enhanced population photosynthetic efficiency and optimized spatial distribution of the leaves of individual plants. This can be seen through high-yielding hexaploid wheat where a moderate leaf angle, leaf area index, and greater light transmittance were beneficial to the lower parts of plant canopy to receive more radiation, and finally enhanced assimilate synthesis [43]–[44]. During the domestication from diploid to tetraploid wheats, the dominant horizontal distribution of canopy structure was replaced by vertical distribution, and it was the difference in canopy structure to enable current commercial cultivars to be distinguished from their wild relatives [9], [45]–[46].

The increase in leaf area from diploid to tetraploid and further to hexaploid wheat was presumably reliable on the availability of carbon and nitrogen resources [47]–[48]. However, leaf net photosynthetic rate was decreased from diploid to tetraploid wheat, but increased from tetraploid to hexaploid. This phenomenon was caused by the difference in canopy stances among wheat species. Canopy stances were shifted from compact type of diploid wheats to incompact type of tetraploid wheats, and further to more compact type of hexaploid wheats. It suggested that neither natural selection and early domestication nor recent plant breeding practice has increased the availability of carbon assimilation through an increase in leaf net photosynthetic rate to support the increase in leaf area.

During the domestication from tetraploid to hexaploid wheat, the biomass of root system was decreased, presumably because of a reduction in root number and root length at the level of individual plants [49]. Tillering in wheat is closely related to the production of nodal roots and hence affects the biomass size of root system [49]–[50]. This study showed that there was a continuous reduction in the number of tillers from diploid to tetraploid and further to hexaploid wheat. It suggested that the reduction in root biomass was due to a reduction in the number of roots, particularly the nodal roots. It is hypothesized that the reduction in root biomass has lessened the competition of individual plants for water and made high yield and dense planting possible in the process of domestication from diploid to tetraploid, further to hexaploid [50]–[51]. The smaller root systems of tetraploid and hexaploid wheats have led to smaller root-shoot ratio. It was likely that this reduction has also led to increased harvest index [6], [11].

The change in biomass allocation between root and shoot rather than the increase in net leaf photosynthetic rate was an important resource flow in the process of dryland wheat domestication. This resource flow directly influenced the final yield formation of wheat crops [12], [16], [22], [52]. To some extent, root system acted as a provider of carbon assimilates to support the increase in leaf area and stem thickness. Under soil water deficit conditions, wild wheat generally had large root-shoot ratio, which was the result of natural selection and early domestication. However, as a result of recent artificial domestication, a lower root-shoot ratio was required to ensure that more shoot biomass was available for yield formation [6], [53]–[54]. During the domestication of dryland wheats, there was a significant increase in both total and aboveground biomass as well in the distribution of stems, leaves and sheaths. The increase in aboveground biomass was the material basis of canopy construction as tetraploid and hexaploidy wheats had wider leaves, larger individual plants. Consequently, the domestication program brought about well-structured canopy and optimized biomass allocation pattern, allowing more biomass to be distributed into photosynthetic organs.

Compared with tetraploid and hexaploid wheats, diploid wheats had larger root system and greater tiller number, which acted as the assurance for strong individual competition ability [2], [55]. Dryland wheat is grown with the aim of having a high population yield, which requires weak individual competition ability since strong competition between individual plants is always in contradiction to the increase in population yield. It was suggested by Donald (1968) that reducing growth redundancy was beneficial to improve population yield in wheat crops [56]. Massive root system is viewed as typical growth redundancy of crop. The two diploid wheats used in this study had massive growth redundancy as they had high root-shoot ratio and canopy width-height ratio, small spikes and tightly encapsulated seeds. These are typical phenotypic characteristics conferring low population yield in wheat [57]–[58].

Existing studies showed that population yield of wheat crop was closely associated with spike length, plant height, height-spike ratio, first node diameter, position of flag leaf, length-width ratio of penultimate and flag leaves [56], [59]–[60]. Our experimental data provided clear evidence that root biomass of modern wheat was decreased significantly, yet aboveground parts became larger. Individual plants became higher and larger with less tiller number at the same time. Meanwhile, the plants got more compact spatial stance with longer internode below the spike collar and larger cross sectional area of all the internodes. The leaf position of top three leaves was raised upward with enlarged leaf area, but the leaves tended to become shorter and wider.

We therefore proposed a hypothesis that the change in biomass allocation pattern brought about spatial stance pattern of leaf and canopy and accordingly affected leaf photosynthetic rate during the domestication process of dryland wheats. In our study, the change in biomass allocation led to significant difference in morphological characteristics of aboveground part, particularly in leaf spatial architecture. Well-structured plant architecture was beneficial to weaken the intensity of competition among various organs of aboveground part as well as individual plants within a population, and consequently improved population photosynthetic rate. The increases in the internode diameter and stem weight provided a powerful support for hexaploid wheat canopies to bear their big spikes. In present study, leaf type characteristics appeared to be critical experimental evidence supporting the hypothesis of McIntyre et al. (1996) and Brooks et al. (2000) that for high-yielding wheat, moderate leaf angle, greater leaf area index and light transmittance were beneficial to the lower part of canopy to get more light and profiting assimilate synthesis [43]–[44].

Interestingly, our data indicated that the length from the first to the fourth internodes increased, yet the length of the internode under spike was properly shortened. The change in internode length was a self-structured modulation, which guaranteed the increase in plant height without population lodging under dense planting. In our present study, the fifth internode of tetraploid wheat is the longest. It suggested that the change in plant type from tetraploid to modern hexaploid be mainly featured by the variation in canopy structure in the vertical direction. During the process of dryland wheat domestication, individual competition ability of wheat crop to snatch limited water resource in soil tended to be decreased, while canopy structure was optimized to obtain greater population yield.

In the process of dryland wheat domestication from tetraploid to hexaploid, total biomass remained invariable although the biomass of root system was decreased, the aboveground biomass increased. In the meantime, spike biomass increased remarkably while biomass of other organs remained unchanged. Since grain yield in wheat is determined by both total biomass and biomass allocation pattern, its plant type evolution is the result of selection that aims for population yield [14], [28]. Through breeding, root-to-shoot ratio continued to decrease and thereby biomass allocation to aboveground organs increased continuously, in which growth redundancy tended to reduce and the competition among individual plants was decreased [56], [58]–[59], [63]–[64].

Finally, the alteration of biomass allocation caused a change in the source-sink relationship. It was likely that from tetraploid to hexaploid, the source of assimilate synthesis became stronger because of greater leaf area and stronger sheath and stem. Hereby, total grain-leaf ratio, grain-leaf ratio of top three leaves, total spike leaf ratio and the spike-leaf ratio of top three leaves all increased, in which more assimilates were distributed to the sink instead of the source organs. Consequently, the domestication of source-sink relationship of dryland wheat from wild to modern varieties has undergone through two important phases. The first phase was to enhance source dimension, and the second one was to strengthen sink dimension. The two phases were evolutionarily mutually related with the aim to ultimately realize coordinated development of source and sink relationship [8], [61]–[64].

## Conclusions

It can be concluded that the domestication from diploid to tetraploid wheat was a process in which plant canopy became more well-structured by changing biomass allocation from belowground to aboveground. More biomass was distributed into photosynthetic organs because of increased leaf area and enhanced photosynthetic capacity. The increase in leaf area presumably changed the opening angle of top leaves in the canopy because more space was required to accommodate larger leaves. High leaf area index, a moderate leaf angle and improved light transmittance to the lower part of canopy were beneficial in increasing the availability of carbon assimilates. The domestication from tetraploid to modern hexaploid wheat tended to continuously decrease individual competition for underground water and the optimization and adjustment of aboveground canopy structure to realize a high population yield. Under dual pressures of natural selection and artificial selection, root system size of dryland wheat was gradually lessened, while aboveground architecture evolved towards a well-structured canopy.
